# Decreased cortical gyrification and surface area in the left medial parietal cortex in patients with treatment‐resistant and ultratreatment‐resistant schizophrenia

**DOI:** 10.1111/pcn.13482

**Published:** 2022-10-27

**Authors:** Kazutoshi Kitajima, Shunsuke Tamura, Daiki Sasabayashi, Shinichiro Nakajima, Yusuke Iwata, Fumihiko Ueno, Yoshifumi Takai, Junichi Takahashi, Fernando Caravaggio, Wanna Mar, Edgardo Torres‐Carmona, Yoshihiro Noda, Philip Gerretsen, Vincenzo de Luca, Masaru Mimura, Shogo Hirano, Tomohiro Nakao, Toshiaki Onitsuka, Gary Remington, Ariel Graff‐Guerrero, Yoji Hirano

**Affiliations:** ^1^ Department of Neuropsychiatry Graduate School of Medical Sciences, Kyushu University Fukuoka Japan; ^2^ Department of Neuropsychiatry University of Toyama Graduate School of Medicine and Pharmaceutical Sciences Toyama Japan; ^3^ Research Center for Idling Brain Science University of Toyama Toyama Japan; ^4^ Department of Neuropsychiatry School of Medicine, Keio University Tokyo Japan; ^5^ Research Imaging Centre, Centre for Addiction and Mental Health (CAMH) Toronto Ontario Canada; ^6^ Department of Neuropsychiatry University of Yamanashi Faculty of Medicine Yamanashi Japan; ^7^ Department of Neuropsychiatry National Hospital Organization Kyushu Medical Center Fukuoka Japan; ^8^ Department of Psychiatry University of Toronto Toronto Ontario Canada; ^9^ Institute of Industrial Science The University of Tokyo Tokyo Japan

**Keywords:** gyrification, surface area, treatment‐resistant schizophrenia

## Abstract

**Aim:**

Validating the vulnerabilities and pathologies underlying treatment‐resistant schizophrenia (TRS) is an important challenge in optimizing treatment. Gyrification and surface area (SA), reflecting neurodevelopmental features, have been linked to genetic vulnerability to schizophrenia. The aim of this study was to identify gyrification and SA abnormalities specific to TRS.

**Methods:**

We analyzed 3T magnetic resonance imaging findings of 24 healthy controls (HCs), 20 responders to first‐line antipsychotics (FL‐Resp), and 41 patients with TRS, including 19 clozapine responders (CLZ‐Resp) and 22 FL‐ and clozapine‐resistant patients (patients with ultratreatment‐resistant schizophrenia [URS]). The local gyrification index (LGI) and associated SA were analyzed across groups. Diagnostic accuracy was verified by receiver operating characteristic curve analysis.

**Results:**

Both CLZ‐Resp and URS had lower LGI values than HCs (*P* = 0.041, Hedges *g* [*g*_H_] = 0.75; *P* = 0.013, *g*_H_ = 0.96) and FL‐Resp (*P* = 0.007, *g*_H_ = 1.00; *P* = 0.002, *g*_H_ = 1.31) in the left medial parietal cortex (Lt‐MPC). In addition, both CLZ‐Resp and URS had lower SA in the Lt‐MPC than FL‐Resp (*P* < 0.001, *g*_H_ = 1.22; *P* < 0.001, *g*_H_ = 1.75). LGI and SA were positively correlated in non‐TRS (FL‐Resp) (*ρ* = 0.64, *P* = 0.008) and TRS (CLZ‐Resp + URS) (*ρ* = 0.60, *P* < 0.001). The areas under the receiver operating characteristic curve for non‐TRS versus TRS with LGI and SA in the Lt‐MPC were 0.79 and 0.85, respectively. SA in the Lt‐MPC was inversely correlated with negative symptoms (*ρ* = −0.40, *P* = 0.018) and clozapine plasma levels (*ρ* = −0.35, *P* = 0.042) in TRS.

**Conclusion:**

LGI and SA in the Lt‐MPC, a functional hub in the default‐mode network, were abnormally reduced in TRS compared with non‐TRS. Thus, altered LGI and SA in the Lt‐MPC might be structural features associated with genetic vulnerability to TRS.

Schizophrenia is a debilitating neuropsychiatric disorder, the underlying pathological mechanisms of which remain unclear.^1^, ^2^ Although antipsychotic drugs targeting dopaminergic neural transmission have been mainly used to treat schizophrenia, approximately one third of patients with schizophrenia show little response to first‐line medications; this drug‐resistant condition is generally defined as treatment‐resistant schizophrenia (TRS).^3^, ^4^, ^5^ More specifically, patients with schizophrenia can be classified into three groups based on their response to antipsychotics.^6^, ^7^ The first group (responders to first‐line antipsychotics [FL‐Resp]) responds to one or more typical or atypical antipsychotics (i.e. first‐line antipsychotics), the second group (clozapine responders [CLZ‐Resp]) responds to clozapine (which is an antipsychotic that may have a different mechanism from first‐line antipsychotics)^8^ but not to first‐line antipsychotics, and the third group (patients with ultratreatment‐resistant schizophrenia [URS]) does not respond to clozapine or first‐line antipsychotics. In general, the TRS is composed of CLZ‐Resp and URS. Approximately 40% and 60% of TRS are known to be CLZ‐Resp and URS, respectively.^5^ The pathophysiological mechanisms underlying schizophrenia may differ among these groups, yet this remains to be elucidated.^9^, ^10^

Several neuroimaging studies have explored brain abnormalities characterized by TRS using various modalities, such as magnetic resonance imaging (MRI), magnetic resonance spectroscopy (MRS), diffusion tensor imaging, and positron emission tomography.^10^, ^11^, ^12^ These studies have reported biochemical features of TRS, such as low dopamine synthesis in the striatum^13^, ^14^, ^15^ and high glutamatergic neurometabolite levels in the medial prefrontal cortex and the anterior cingulate cortex (ACC).^9^, ^16^, ^17^ Notably, Iwata *et al*.^9^ assessed glutamate and glutamate + glutamine levels in patients with URS using MRS and found that abnormal (higher) glutamatergic neurometabolite levels in the dorsal ACC were specific to URS.

On the other hand, there are a limited number of studies that have identified brain structural characteristics unique to TRS and URS. Several studies have reported altered cortical and subcortical structures in TRS compared with non‐TRS (i.e. FL‐Resp), such as reduced cortical thickness in the left dorsolateral prefrontal cortex,^18^ decreased volume of lateral medial frontal and lateral temporal regions,^19^ and lower fractional anisotropy measured as white matter integrity in widespread tracts.^20^ However, among these studies, the brain region and structure metric abnormalities specific to TRS have little in common.^10^ Knowledge concerning structural features specific to URS is also limited, although several studies have attempted to classify TRS into CLZ‐Resp and URS based on cortical thickness,^21^ subcortical volume,^22^ and fractional anisotropy.^23^ Thus, further investigations designed to compare TRS (CLZ‐Resp and URS) and non‐TRS (FL‐Resp) are needed to clarify the brain structures underlying TRS. Given that cortical surface abnormalities are prominent and different depending on the stage or the severity of the disease,^24^ it should be important to focus on cortical surface structure metrics such as cortical gyrification and cortical surface area (SA).

Cortical gyrification is a morphological feature that indicates the developmental process of cortical folding patterns.^25^, ^26^ In general, gyrification is defined as the ratio of the total area of the outer surface of the cortex to the superficially exposed portion of the outer surface and is quantified as the gyrification index (GI).^26^ Recently, the degree of gyrification was quantified by the local gyrification index (LGI), which is methodologically superior to the conventional GI method because it takes into account the inherent three‐dimensional nature of the cortical surface.^27^ Gyrification begins to develop around the early second trimester of pregnancy and ends in the third trimester, with relatively little change in GI in healthy populations thereafter.^28^ Therefore, evaluating gyrification characteristics has the potential to identify neurodevelopmental brain deficits.^29^, ^30^

Gyrification abnormalities in patients with schizophrenia have been extensively studied, although anomalous patterns of gyrification vary depending on the different clinical characteristics of patients with schizophrenia.^31^, ^32^, ^33^, ^34^ Among them, it has been reported that reduced GI values around the precuneus and posterior cingulate cortex were observed not only in patients with schizophrenia^32^, ^35^, ^36^, ^37^ and patients with first‐episode psychosis^38^ but also unaffected relatives of patients with schizophrenia with high genetic load.^39^ In addition, Docherty *et al*.^40^ have suggested that gyrification is highly heritable and has a strong phenotypic and genetic association with cortical SA. Thus, the decrease in gyrification and SA might be attributable to genetic vulnerability associated with the early neurodevelopmental abnormalities that characterize schizophrenia. Recently, the existence of polygenes specific to TRS has been suggested,^41^ but no studies have examined gyrification and SA in patients with TRS.

Given these pieces of evidence, examining gyrification and its associated SA in individuals with schizophrenia based on their response to antipsychotics is of great importance and may shed light on understanding TRS pathology. The aim of this study was to identify the brain structural features specific to TRS or URS by comparing gyrification and SA between FL‐Resp, CLZ‐Resp, URS, and healthy controls (HCs). The degree of gyrification was quantified by the LGI. We also analyzed the SA in the brain regions where abnormal LGI specific to TRS was observed and examined its relationship with LGI. Notably, Palaniyappan *et al*.^38^ showed that patients who responded poorly to treatment for psychosis, including schizophrenia, had reduced GI values in several frontotemporal regions as well as the insula, the precuneus, the angular gyrus, and the lingual gyrus at the onset of the first episode. Based on this finding, we hypothesized that reductions in GI and its associated SA would be observed within these regions, specifically in patients with TRS (CLZ‐Resp and URS).

## Methods

The protocol for the research project was approved by the research ethics board at the Centre for Addiction and Mental Health (CAMH), which conform to the provisions of the Declaration of Helsinki. All participants received explanations about the procedures involved in this research and gave informed consent before participation. Data from this study were obtained from a cross‐sectional MRS study that examined glutamatergic neurometabolite levels in URS.^9^

### Participants

The final sample of participants consisted of 24 HCs (age, 41.5 ± 13.5 years; 18 men), 20 FL‐Resp (age, 43.8 ± 12.6 years; 15 men), 19 CLZ‐Resp (age, 43.1 ± 13.8 years; 13 men) and 22 URS (age, 45.9 ± 11.9 years; 18 men). The participants were aged 18 years or older at the time of MRI scanning. The patient groups and HC group were matched in terms of age and sex. All participants received explanations about the procedures involved in this research and gave informed consent before participation. All patients met the *DSM‐IV* criteria for schizophrenia based on the Structured Clinical Interview for *DSM‐IV*. The criteria for FL‐Resp, CLZ‐Resp, and URS are described below. HCs were screened using the Mini‐International Neuropsychiatric Interview and had neither a history of psychiatric illness nor neurological disorders, including epilepsy. The exclusion criteria for all groups were as follows: (1) substance abuse or dependence during the past 6 months; (2) positive urine drug screen at inclusion or prior to MRI scanning; (3) neurological illness; (4) head trauma with loss of consciousness for more than 30 min; (5) any contraindications of MRI, such as implanted metal in the body, tattoos, pregnancy, and claustrophobia; and (6) inability to provide informed consent.

Antipsychotic treatment resistance and responsiveness were defined by the modified Treatment Response and Resistance in Psychosis (TRRIP) Working Group Consensus criteria.^6^ In the current study, specific criteria of treatment resistance were as follows: Clinical Global Impression Severity (CGI‐S) score ≥4 (moderate) and a score ≥4 (moderate) for two positive symptom items from the Positive and Negative Syndrome Scale (PANSS).^6^, ^7^, ^13^ Conversely, specific criteria for treatment response were CGI‐S score ≤3, scores on all positive symptom items from the PANSS ≤3, and no symptomatic relapse within the previous 3 months.^7^, ^13^

The FL‐Resp were taking an antipsychotic, but not clozapine, for >6 consecutive weeks and met the criteria of treatment response. The FL‐Resp had no history of treatment resistance. The CLZ‐Resp were taking clozapine for >6 weeks and were treatment responsive after a history of treatment resistance to at least two other antipsychotics where chlorpromazine antipsychotic dose equivalents of >400 mg were administered for >6 consecutive weeks. The URS showed resistance to clozapine treatment after experiencing resistance to at least two other antipsychotics in the same manner as the CLZ‐Resp. They were treated with >300 mg/day clozapine for >6 weeks.

### Magnetic resonance imaging

The participants were scanned using a 3T scanner (Discovery MR750, GE Healthcare, Waukesha, WI, USA) equipped with an 8‐channel head coil at the CAMH. A three‐dimensional inversion recovery–prepared T1‐weighted MRI scan was conducted using the GE brain volume (BRAVO) sequence (echo time = 3.00 ms, repetition time = 6.74 ms, inversion time = 650 ms, flip angle = 8, field of view = 23 cm, 256 × 256 matrix, slice thickness = 0.9 mm).

### Image processing

We used FreeSurfer software (version 6.0.0; https://surfer.nmr.mgh.harvard.edu/) for preprocessing, LGI and SA analysis, and whole‐brain statistical analysis. T1‐weighted MRI images were preprocessed with the standard automatic reconstruction algorithm of FreeSurfer, which included skull stripping, volumetric labeling, intensity normalization, white matter segmentation, cortical surface atlas registration, surface extraction, and gyral labeling. The quality of the reconstructed images was visually checked by a trained researcher (K. K.) who was blinded to the demographic information of the participants. If there were any inaccuracies in brain tissue segmentation, the researcher manually edited them. After manual editing, 2 HCs, 1 FL‐Resp, 5 CLZ‐Resp, and 2 URS participants were excluded from further analysis because accurate separation between gray matter and white matter was not possible, resulting in 24 HCs, 20 FL‐Resp, 19 CLZ‐Resp, and 22 URS participants. We quantified the individual vertexwise LGI values of the entire cortex using the method of Schaer *et al*.^27^ The detailed procedure of the LGI analysis was as follows: First, the outer surface, which enveloped the hemisphere and tightly wrapped the pial surface, was created. Second, spherical regions of interest (ROIs) on the outer surface and their corresponding patches on the pial surface were estimated. Approximately 800 ROIs (radius = 25 mm) covering the entire cortex and overlapping partly with each other were created with the vertex as the center point on the hull surface. The individual vertexwise LGI values were mapped on a standard template image (fsaverage) and smoothed with a three‐dimensional 5‐mm full width at half maximum Gaussian kernel.

### Statistical analysis

We conducted one‐way ANOVA and *χ* ^2^ tests with group as a between‐patient factor to assess group differences in the demographic variables. Unpaired *t* tests and *χ* ^2^ tests with multiple comparison correction (Bonferroni correction with an overall *α* level of 0.05 were applied) were performed to test the differences and the ratios between each pair of groups.

In the whole‐brain statistical analysis using a general linear model, a contrast matrix was set up to estimate a main effect of the group for LGI in each vertex. In addition, we also used the contrast matrixes to compare the LGI in each vertex between each pair of groups, between all patients with schizophrenia (FL‐Resp + CLZ‐Resp + URS) and HCs, between TRS (CLZ‐Resp + URS) and FL‐Resp, and between TRS (CLZ‐Resp + URS) and HCs. Regarding the use of covariates, Hyatt *et al*.^42^ suggested that inclusion of demographic variables of the participants in a regression model should be considered carefully because it might alter the significance of the findings by removing the meaningful variance from the other predictors. We matched age and sex between groups in the present study as described above, and, therefore, we used a general linear model without any covariates for the whole‐brain statistical analysis. A permutation simulation was used to perform clusterwise correction for multiple comparisons, with a cluster‐forming threshold of *P* < 0.001. We performed a total of 1000 simulations for each comparison using a threshold of *P* < 0.05 to define significant clusters.

We regarded the cluster in which a significant main effect of the group was observed as an ROI, and then the individual values of the LGI and SA were extracted from the ROI. The extracted values were submitted to one‐way ANOVA with group (HCs, FL‐Resp, CLZ‐Resp, and URS) as a between‐patient factor using R software (version 4.0.3; http://cran.r-project.org/). Unpaired *t* tests with multiple comparison corrections using the Bonferroni method were performed to compare the differences in the LGI and SA between each pair of groups. The effect sizes are expressed as *g*_H_.

Based on the results of vertexwise and ROI analyses (see Results), further statistical analyses of LGI and SA were conducted separately. Comparison groups were divided into HCs, non‐TRS (FL‐Resp), and TRS (CLZ‐Resp and URS) groups because there was no significant difference between CLZ‐Resp and URS. The relationships between LGI and SA were examined in HCs, non‐TRS, and TRS groups using Spearman rank correlation coefficients. We also examined Spearman correlations of the extracted values of the LGI and SA with clinical measures (i.e. antipsychotic dose based on chlorpromazine antipsychotic dose equivalents, clozapine and norclozapine plasma levels in TRS, age at initial MRI scan, and PANSS scores) in the non‐TRS and TRS groups. For statistical tests of the correlation coefficients, a Bonferroni correction was applied to the *P*‐values according to the number of examinations for each combination of cortical metrics and clinical measures.

The optimal sensitivity and specificity of the discriminant value of LGI or SA to differentiate between TRS and non‐TRS were determined via receiver operating characteristic (ROC) curve analysis using a nonparametric approach. In addition to each LGI or SA, we also conducted ROC curve analysis with the combination of LGI and SA as discriminant values. We calculated the Youden index for each cutoff value ([sensitivity + specificity] – 1) to find the cutoff values that maximized the discriminating power. We compared statistically area under the curves (AUCs) of the three ROC curves.^43^ Bonferroni correction was applied to the *P*‐values according to the number of tests.

## Results

### Demographic and clinical characteristics

The detailed demographic and clinical characteristics are described in Table 1. We found significant main effects of group on education years, cigarette smoking, and PANSS, Brief Psychiatric Rating Scale (BPRS), CGI‐S, and Global Assessment of Functioning (GAF) scores, while there were no significant main effects of group on age, sex, age at illness onset, illness duration, or chlorpromazine antipsychotic dose equivalents. Multiple comparisons showed that the HCs group had more years of education and a lower rate of cigarette smoking than the other groups. The URS group had higher PANSS total, PANSS subscale, BPRS, and CGI‐S scores than the CLZ‐Resp and FL‐Resp groups. These results indicate that the symptoms of the URS were more severe than those of the CLZ‐Resp and FL‐Resp. The URS also had lower GAF scores than the CLZ‐Resp and FL‐Resp.

**Table 1:** Demographic and clinical characteristics of the study groups

	HCs (*n* = 24)	FL‐Resp (*n* = 20)	CLZ‐Resp (*n* = 19)	URS (*n* = 22)	*χ* ^2^, *F*	df	*P*‐value	Multiple comparisons
Age (years)	41.5 (13.5)	43.8 (12.6)	43.1 (13.8)	45.9 (11.9)	0.45	3, 81	0.717	
Men/women (n)	18/6	15/5	13/6	18/4	0.99	3	0.804	
Education (years)	15.7 (2.1)	13.2 (2.5)	12.9 (3.3)	12.3 (2.5)	7.43	3, 79	<0.001	HC > FL‐Resp HC > CLZ‐Resp HC > URS
Cigarette smoking (use/no use)	1/23	13/7	8/10	10/11	18.88	3	<0.001	HC < FL‐Resp HC < CLZ‐Resp HC < URS
Onset age (years)		24.9 (6.3)	23.9 (6.8)	21.7 (4.9)	1.51	2, 56	0.230	
Illness duration (years)		20.0 (12.5)	18.4 (12.6)	23.8 (12.6)	0.95	2, 56	0.394	
Chlorpromazine antipsychotic dose equivalents (mg/day)		466.8 (232.2)	441.2 (253.9)	556.2 (221.6)	1.38	2, 58	0.261	
Clozapine dose (mg/day)			294.1 (169.3)	371.6 (147.0)	2.46	1, 39	0.125	
Clozapine plasma levels (nmol/L)			1522.9 (1160.7)	2212.2 (1276.3)	2.69	1, 32	0.111	
Norclozapine plasma levels (nmol/L)			987.3 (637.0)	1337.2 (702.4)	2.29	1, 32	0.140	
Clozapine/norclozapine plasma level ratio			1.49 (0.50)	1.65 (0.49)	0.88	1, 32	0.356	
PANSS total		56.5 (9.5)	55.3 (10.5)	83.4 (11.8)	46.53	2, 58	<0.001	FL‐Resp < URS CLZ‐Resp < URS
PANSS positive		10.8 (2.2)	11.2 (1.9)	22.8 (3.9)	118.50	2, 58	<0.001	FL‐Resp < URS CLZ‐Resp < URS
PANSS negative		15.8 (3.5)	16.1 (4.3)	20.5 (4.4)	8.51	2, 58	<0.01	FL‐Resp < URS CLZ‐Resp < URS
PANSS general		30.0 (4.9)	28.5 (6.1)	40.2 (7.4)	21.64	2, 58	<0.001	FL‐Resp < URS CLZ‐Resp < URS
BPRS		40.4 (6.4)	38.7 (5.8)	57.9 (9.8)	40.59	2, 58	<0.001	FL‐Resp < URS CLZ‐Resp < URS
GAF		63.8 (3.0)	66.3 (7.3)	43.2 (10.5)	54.86	2, 56	<0.001	FL‐Resp < URS CLZ‐Resp < URS
CGI‐S		2.7 (0.5)	2.9 (0.3)	4.7 (0.8)	78.27	2, 56	<0.001	FL‐Resp < URS CLZ‐Resp < URS
Antipsychotic medications								
Clozapine		0	19	22				
Flupenthixol		2						
Fluphenazine		1						
Haloperidol		1						
Loxapine		1						
Olanzapine		7						
Paliperidone		2						
Perphenazine		1						
Risperidone		4						
Ziprasidone		1						

The data are given as mean (SD). Bonferroni corrections with an overall α level of 0.05 were applied to account for multiple comparisons.

BPRS, Brief Psychiatric Rating Scale; CGI‐S, Clinical Global Impression Severity scale; CLZ‐Resp, clozapine responders; FL‐Resp, first‐line antipsychotic responders; GAF, Global Assessment of Functioning; HCs, healthy controls; PANSS, Positive and Negative Syndrome Scale; URS, ultratreatment‐resistant schizophrenia.

### Group differences in LGI

**Fig. 1:**
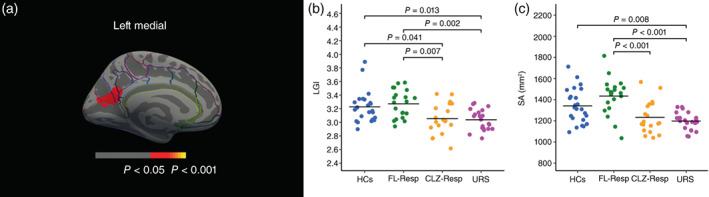
Decreased cortical gyrification and surface area (SA) in the left medial parietal cortex (Lt‐MPC) in both clozapine responders (CLZ‐Resp) and ultratreatment‐resistant schizophrenia (URS) compared with healthy controls (HCs) and responders to first‐line antipsychotics (FL‐Resp). (a) Clusters showing a significant main effect of group on the local gyrification index (LGI). The maps are shown for the left hemispheres in the medial view. The horizontal bar shows cluster *P*‐value. (b) Comparison of the LGI in the Lt‐MPC between the four groups (CLZ‐Resp, FL‐Resp, HCs, and URS). (c) Comparison of the SA in the Lt‐MPC between the four groups.

A whole‐brain statistical analysis of LGI values revealed that there was a significant main effect of group in multiple regions of the medial part of the left hemisphere. We examined the anatomical parcellation of these regions based on the Destrieux atlas in FreeSurfer^44^ and found that there was a significant main effect of group on LGI values in the left default‐mode network (DMN)–related functional hub regions,^45^ namely in the left medial parietal cortex (Lt‐MPC), composed of the left precuneus, parieto‐occipital sulcus, cuneus, calcarine sulcus, subparietal sulcus, posterior‐dorsal part of the cingulate gyrus, and posterior‐ventral part of the cingulate gyrus (Fig. 1a and Table 2). In addition, we found that the URS had significantly lower LGI values in the Lt‐MPC than the FL‐Resp (Fig. S1a and Table S1a) and that the TRS (CLZ‐Resp + URS) had significantly lower LGI values in the Lt‐MPC than non‐TRS (FL‐Resp) (Fig. S1b and Table S1b). There were no significant clusters in comparisons between the other group combinations.

**Table 2:** Clusters with a significant main effect of the group in local gyrification index

	MNI coordinates			Clusterwise	
Cluster size (mm^2^)	*x*	*y*	*z*	*P‐*value	Anatomical region
1025	−5.7	−59.3	21.2	0.01395	Left precuneus, parieto‐occipital sulcus, cuneus, calcarine sulcus, subparietal sulcus, posterior‐dorsal part of the cingulate gyrus, posterior‐ventral part of the cingulate gyrus

MNI, Montreal Neurological Institute.

One‐way ANOVA on the LGI values in the Lt‐MPC extracted from an ROI (Fig. 1b) showed a significant main effect of group (*F* [3, 81] = 7.23, *P* < 0.001). The post hoc tests with Bonferroni correction revealed that the CLZ‐Resp had a significantly lower LGI value in the Lt‐MPC than the HCs (*g*_H_ = 0.75, *P* = 0.041; Bonferroni corrected) and the FL‐Resp (*g*_H_ = 1.00, *P* = 0.007; Bonferroni corrected). The URS also had significantly lower LGI values in the Lt‐MPC than the HCs (*g*_H_ = 0.96, *P* = 0.013; Bonferroni corrected) and FL‐Resp (*g*_H_ = 1.31, *P* = 0.002; Bonferroni corrected). There were no significant differences between the HCs and FL‐Resp (*g*_H_ = 0.20, *P* = 1.00; Bonferroni corrected) or between the CLZ‐Resp and URS (*g*_H_ = 0.09, *P* = 1.00; Bonferroni corrected).

### Group differences in SA

One‐way ANOVA on the SA extracted from the ROI (Fig. 1c) showed a significant main effect of group (*F* [3, 81] = 11.24, *P* < 0.001). The post hoc tests with Bonferroni correction revealed that the CLZ‐Resp had a significantly lower SA value in the Lt‐MPC than the FL‐Resp (*g*_H_ = 1.22, *P* < 0.001; Bonferroni corrected). The URS also had a significantly lower SA value in the Lt‐MPC than the HCs (*g*_H_ = 1.08, *P* = 0.008; Bonferroni corrected) and FL‐Resp (*g*_H_ = 1.75, *P* < 0.001; Bonferroni corrected). There were no significant differences between the HCs and FL‐Resp (*g*_H_ = 0.55, *P* = 0.23; Bonferroni corrected), between the HCs and CLZ‐Resp (*g*_H_ = 0.68, *P* = 0.10; Bonferroni corrected), or between the CLZ‐Resp and URS (*g*_H_ = 0.29, *P* = 1.00; Bonferroni corrected).

### Correlation between LGI and SA

**Fig. 2:**
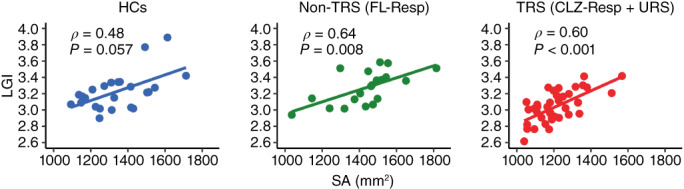
Correlations between local gyrification index (LGI) and surface area (SA). Scatter plots of LGI as a function of SA for healthy control (HCs), nontreatment‐resistant schizophrenia (non‐TRS), or treatment‐resistant schizophrenia (TRS). The non‐TRS corresponds to patients with schizophrenia who responded to first‐line antipsychotics (FL‐Resp), and the TRS is comprised of patients with treatment‐resistant schizophrenia who responded to clozapine (CLZ‐Resp) and patients with ultratreatment‐resistant schizophrenia (URS).

There were significant positive correlations between LGI and SA values in the Lt‐MPC for the non‐TRS (FL‐Resp) (*ρ* = 0.64, corrected *P* = 0.008) and TRS (CLZ‐Resp + URS) (*ρ* = 0.60, corrected *P* < 0.001) (Fig. 2). A marginally significant correlation was also found for the HCs (*ρ* = 0.48, corrected *P* = 0.057).

### ROC curve analysis

**Fig. 3:**
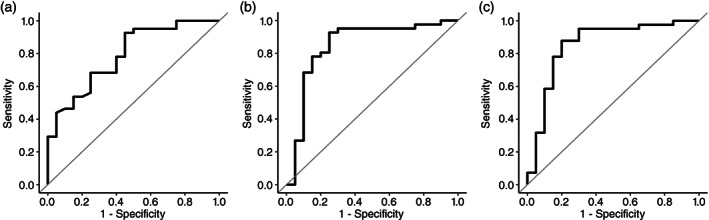
The results of receiver operating characteristic (ROC) curve analysis. The ROC curves between patients with treatment‐resistant schizophrenia (TRS) and patients with schizophrenia who responded to first‐line antipsychotics (non‐TRS) for local gyrification index (a), surface area (b), and their combination (c).

We used ROC curve analyses to explore the discriminant value of the LGI. Figure 3a shows the ROC curve for the LGI between the non‐TRS (FL‐Resp) and TRS (CLZ‐Resp + URS). The AUC of the ROC curve was 0.79 (standard error [SE] = 0.061, *P* < 0.001 [95% CI, 0.67–0.91]), indicating that the discriminant value of LGI could be used to differentiate between the non‐TRS and TRS with moderate accuracy.^46^ The Youden index indicated a favorable cutoff point of 3.29, which resulted in 92.7% sensitivity and 55.0% specificity. Figure 3b shows the ROC curve for the SA between the non‐TRS and TRS. The AUC of the ROC curve was 0.85 (SE = 0.064, *P* < 0.001 [95% CI, 0.72–0.97]), indicating that the discriminant value of SA could be used to differentiate between the non‐TRS and TRS with excellent accuracy. The Youden index indicated a favorable cutoff point of 1368.50, which resulted in 92.7% sensitivity and 75.0% specificity. Figure 3c shows the ROC curve for the combination of LGI and SA between the non‐TRS and TRS. The AUC of the ROC curve was 0.85 (SE = 0.061, *P* < 0.001 [95% CI, 0.73–0.97]), indicating that the discriminant value of SA could be used to differentiate between the non‐TRS and TRS with excellent accuracy. The Youden index indicated a favorable cutoff point of 0.606, which resulted in 87.8% sensitivity and 80.0% specificity. There were no significant differences of AUCs between the LGI and SA (*Z* = 1.01, *P* = 0.93; Bonferroni corrected), between the LGI and the combination of LGI and SA (*Z* = 1.26, *P* = 0.62; Bonferroni corrected), or between the SA and the combination of LGI and SA (*Z* = 0.58, *P* = 1.00; Bonferroni corrected).

### Correlation of LGI and SA with clinical measures

**Fig. 4:**
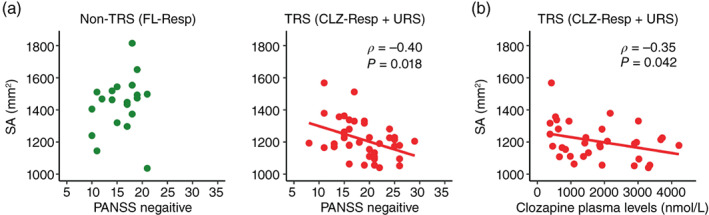
Correlations of surface area (SA) with clinical variables. (a) Scatter plots of SA in the left medial parietal cortex (Lt‐MPC) as a function of negative score in the Positive Negative Syndrome Scale (PANSS) for nontreatment‐resistant schizophrenia (non‐TRS) or treatment‐resistant schizophrenia (TRS). The non‐TRS corresponds to patients with schizophrenia who responded to first‐line antipsychotics (FL‐Resp), and the TRS is comprised of patients with treatment‐resistant schizophrenia who responded to clozapine (CLZ‐Resp) and patients with ultratreatment‐resistant schizophrenia (URS). (b) Scatter plots of SA in the Lt‐MPC as a function of clozapine plasma levels in TRS.

We found no significant correlations of LGI values in the Lt‐MPC with any clinical variables in either the non‐TRS (FL‐Resp) or TRS (CLZ‐Resp + URS) groups. Regarding the SA, there was a significant negative correlation between SA in the Lt‐MPC and PANSS negative scores in the TRS (*ρ* = −0.40, corrected *P* = 0.018) but not in the non‐TRS (*ρ* = 0.25, corrected *P* = 0.56) (Fig. 4a). There was also a negative correlation between the SA value and clozapine plasma levels in the TRS (CLZ‐Resp + URS) (*ρ* = −0.35, *P* = 0.042) (Fig. 4b). No significant correlations between the SA value and the other clinical variables were observed in either the non‐TRS or TRS groups.

## Discussion

The current study indicates that the LGI in the Lt‐MPC, composed of the left precuneus, posterior cingulate gyrus, and parieto‐occipital sulcus, was decreased in both the CLZ‐Resp and the URS compared with the HCs and FL‐Resp. The SA in the same region was decreased in both the CLZ‐Resp and the URS compared with the FL‐Resp. Significant positive correlations between LGI and SA were also observed in the non‐TRS (FL‐Resp) and TRS (CLZ‐Resp + URS). The ROC analyses revealed that LGI and SA values in the Lt‐MPC could differentiate the TRS (CLZ‐Resp + URS) from the non‐TRS (FL‐Resp) with moderate and excellent accuracy, respectively. Regarding clinical features, SA values in the Lt‐MPC were correlated with the severity of negative symptoms in patients with TRS. Given that gyrification patterns and their relations with the SA reflect neurodevelopmental processes or characteristics associated with genetic vulnerability,^25^, ^26^, ^40^ the present results suggest that decreased gyrification and SA in the Lt‐MPC may represent a neurodevelopmental pathology underpinning treatment resistance in schizophrenia. The current study is the first to find decreased LGI and SA in TRS and therefore provides new insights into the neurobiological mechanism underlying the therapeutic response to antipsychotics.

We found abnormal gyrification unique to TRS in the Lt‐MPC. The decrease gyrification in the Lt‐MPC was also observed in a limited number of previous studies that reported decreased LGI values in multiple regions, including the Lt‐MPC in schizophrenia.^36^, ^37^ However, in most previous studies of schizophrenia, altered gyrification was found in different brain regions, such as the frontal,^31^, ^32^, ^34^, ^47^, ^48^, ^49^, ^50^, ^51^ temporal,^32^, ^34^, ^51^ parietal,^32^, ^34^, ^47^, ^51^, ^52^ and occipital regions.^32^, ^47^, ^51^, ^52^ Given the evidence as a whole, it is conceivable that gyrification deficits in the Lt‐MPC might be a feature of TRS in particular rather than schizophrenia in general.

Regarding gyrification characteristics for TRS, a previous longitudinal study^38^ showed that patients with treatment‐resistant psychosis had lower LGI values than HCs in a wide range of brain regions, including the Lt‐MPC, at the onset of the first episode. Notably, there were no such differences between treatment responders and HCs. On the other hand, in the present study, lower gyrification was confined to the Lt‐MPC in individuals with TRS. Considering that the patients with affective psychosis also participated in addition to patients with schizophrenia in the previous study, the decreased gyrification in the Lt‐MPC might be specific to TRS. In addition, most patients in the current study were in the chronic stage, while the previous study focused on first‐episode psychosis, although reduced gyrification in the Lt‐MPC was common to the two studies. This indicates that reduced gyrification in this region would be a structural feature characterized by TRS regardless of disease stage.

While the present study found hypogyrification in TRS, several schizophrenia studies have reported hypergyrification.^31^, ^33^, ^47^, ^48^, ^51^ A recent systematic review of brain gyrifications in major psychiatric disorders suggested that the illness stage at MRI measurement may result in different patterns of gyrification deficits (hypergyrification or hypogyrification) in schizophrenia.^53^ Specifically, the gyrification of the frontal and temporal regions increased in the early (3 years) or middle illness stage (3–10 years) but decreased in the late stage (>10 years). However, there are no reports regarding the hypergyrification in the parietal and occipital regions, including the precuneus, in any illness stage. Thus, we consider that the gyrifications in the frontal and temporal regions would reflect the states of patients, such as the illness stage and symptom severity. On the other hand, those in the parietal and occipital regions might be more like trait markers reflecting genetic vulnerability, as described previously.^39^

In the course of brain development, the brain undergoes several maturational events to establish neural networks,^54^ and developmental perturbations in the organization of structural and functional networks can result in various psychiatric disorders, including schizophrenia.^55^, ^56^, ^57^ Considering that the overall pattern of cortical gyrification is established within a few years after birth and remains consistent for most of the lifespan,^28^ and from the perspective of abnormal neurodevelopment pathology of schizophrenia,^29^, ^30^, ^58^, ^59^ perturbations in the cortical gyrification pattern may underlie a certain core pathology of schizophrenia. Importantly, the compact wiring or intricate structure of cortical gyrification is crucial for the brain to maintain its efficient neural networks.^60^, ^61^ Accumulating evidence also indicates a tight relationship between disrupted altered gyrification patterns and functional connectivity of the cerebral cortex in schizophrenia.^62^ Therefore, it is conceivable that the decreased gyrification in the Lt‐MPC would deteriorate the efficiency of structural and functional connections between the Lt‐MPC and the other brain regions within the context of the gyrification‐based structural connectome.^63^ This gyrification‐based structural connectome concept is also important for the prediction of early psychosis. Indeed, Das *et al*.^63^ showed that constructing a gyrification‐based structural connectome may facilitate the prediction of future psychosis in clinical high‐risk patients.

Notably, the most recent perspectives on the DMN are that the left precuneus and posterior cingulate cortex, consisting of the Lt‐MPC region (considered the most densely connected functional hub of the DMN), are the central functional regions for integrating external and internal information, allowing for shared communication and social interaction.^64^ Given that disordered experience of the self (self‐disorder) and disturbances in shared communication and social interaction are prominent in schizophrenia, and these lead to poor outcomes,^65^, ^66^ structural and functional deficits in the Lt‐MPC region could represent neural correlates of these severe self‐related symptoms. Indeed, the literature by Liemburg *et al*.^67^ showed that patients with schizophrenia with poor insight demonstrate decreased connectivity in DMN regions (ACC and precuneus) implicated in self‐related processing. Moreover, Lee *et al*.^68^ reported that poorer clinical outcomes in schizophrenia were associated with decreased DMN connectivity, including the Lt‐MPC region. Taken together, the evidence leads us to speculate that gyrification deficits in the Lt‐MPC underlie the connection of DMN deficits with impaired self‐related processing or self‐disorder, which may, in turn, cause poorer clinical outcomes as seen in TRS. However, the present study analyzed gyrification characteristics only and did not examine the connection between LGI and self‐related processing. Therefore, further research is needed to confirm the direct relationship among functional and structural aspects of the Lt‐MPC, self‐disorder symptoms, and its clinical outcome in TRS.

Importantly, LGI deficit was also accompanied by decreased SA unique to TRS in the Lt‐MPC. Decreased SA was also observed in previous studies, showing a decreasing trend of SA values in schizophrenia.^34^, ^69^, ^70^ While a limited number of studies failed to find SA deficits between TRS and non‐TRS,^71^, ^72^ the current study is the first to find decreased SA in the Lt‐MPC, especially in TRS. The result of the correlational analysis between LGI and SA is consistent with a previous finding showing their positive correlation^73^ and supports the finding that gyrification had a strong phenotypic and genetic association with cortical SA.^40^ Considering that SA deficits in the precuneus are related to ZNF804A rs1344706, a prominent susceptibility gene for schizophrenia,^74^ the current results indicate that not only LGI but also SA in the Lt‐MPC is a brain structural characteristic reflecting genetic vulnerability in TRS.

We found an inverse correlation between the SA value in the Lt‐MPC and PANSS negative scores in TRS, whereas there were no such correlations shown in non‐TRS (FL‐Resp) (Fig. 4a). These results indicate that neurodevelopmental structural features in the Lt‐MPC might underlie negative symptoms of TRS. Regarding the patients in FL‐Resp (non‐TRS), the neurodevelopmental structural features in FL‐Resp were thought to be within the normal range because SA values were not significantly different between the HCs and FL‐Resp. Therefore, it is conceivable that the individual differences in the SA in FL‐Resp (non‐TRS) were not connected to clinical symptoms. It should also be noted that it is difficult to interpret group differences in the correlation between the SA value and negative symptoms because non‐TRS and TRS are classified based on positive symptoms. The present findings suggest the necessity of reconsidering the classification criteria of TRS. In addition to positive symptoms, negative and cognitive symptoms also have a very large impact on treatment resistance.^6^, ^75^ In fact, a recent review regarding negative symptoms in schizophrenia also pointed out that negative symptoms generally do not respond well to currently available antipsychotic treatment.^76^ We also found an inverse correlation between the SA value in the Lt‐MPC and clozapine plasma levels in TRS. It has been reported that higher clozapine plasma levels would be necessary to improve clinical symptoms in a set of patients.^77^ Thus, patients with more severe neurodevelopmental abnormalities in the Lt‐MPC might be inclined to require higher clozapine plasma levels for sufficient treatment.

Notably, one of the current study's objectives was to detect brain structures that can predict the degree of treatment resistance within schizophrenia, allowing for a therapeutic strategy of early clozapine intervention. Our ROC analysis revealed that the LGI and SA values in the Lt‐MPC could differentiate between TRS and non‐TRS with moderate (AUC = 0.79) and excellent (AUC = 0.85) accuracies, respectively. In addition, the combination of LGI and SA could differentiate them with excellent accuracies (AUC = 0.85) (Fig. 3). These results indicate that LGI and its associated SA within the Lt‐MPC may allow us to differentiate at least TRS (CLZ‐Resp and URS) from non‐TRS (FL‐Resp) as a potential neuroimaging marker, although the generalizability of ROC results should be investigated with a larger sample size. From a clinical perspective, this finding is critical since, thus far, there are no such established biomarkers to distinguish between the two.

Some caveats of the current study should be noted. First, although we included the expected number of total participants (n = 95), the number of participants included in each group may be relatively small. Further study with a larger sample size is needed to confirm our results toward clinical application. Second, since this is a study regarding TRS, all of the patients were taking antipsychotics at the MRI scan, and therefore, we could not completely exclude the possible influence of antipsychotics on the results reported here. In particular, it cannot be completely denied that clozapine intake could result in different LGI between FL‐Resp and TRS (CLZ‐Resp and URS). Although it may be challenging, recruiting patients with TRS who were not prescribed clozapine would be needed to solve this limitation in future studies. Third, in previous studies, including the current study, there has been great heterogeneity in the definitions of TRS and URS, and the implementation of guidelines has been inadequate.^78^ Future studies should be performed with standardized definitions. Fourth, one might consider that the current failure to respond to a particular medication scheme might, to some extent, change throughout the course of schizophrenia. We adopted the modified TRRIP Working Group Consensus criteria^6^ to classify patients with schizophrenia in terms of response to treatment at the time of MRI measurement. Therefore, we could not examine LGI and SA taking into account the variable state of unresponsiveness to specific pharmacotherapies. Fifth, although we were able to differentiate TRS (CLZ‐Resp + URS) as a whole from non‐TRS (FL‐Resp), we could not differentiate CLZ‐Resp from URS using LGI and SA. Future studies may need to establish neuroimaging markers that allow us to distinguish these two, as it is also critical to obtain an objective indicator when considering a therapeutic strategy within TRS. Finally, although we discussed cortical gyrification deficits in relation to brain networks and their functional implications, we did not examine the functional aspect of gyrification. Future studies are warranted to investigate the relationship between cortical gyrification and brain activity by conducting multimodal functional imaging.

## Conclusion

The current results demonstrate that the LGI and its associated SA in the Lt‐MPC, a functional hub region of the DMN, were abnormally decreased in patients with TRS (i.e. CLZ‐Resp and URS) compared with non‐TRS (FL‐Resp). The ROC analysis also revealed that the LGI and SA values in the Lt‐MPC could differentiate between the TRS and non‐TRS with moderate and excellent accuracies, respectively. Thus, the altered LGI in the Lt‐MPC might be a potential structural marker that could assist in predicting patients' response to first‐line antipsychotics,^79^, ^80^ although it should be noted that the brain pathologies of TRS and non‐TRS might be neither homogeneous nor mutually exclusive. The present findings warrant longitudinal studies using multimodal functional imaging to elucidate the underlying mechanisms of gyrification in TRS.

## Conflict of Interest

G.R. has received grants from HLS Therapeutics, Inc. F.U. has received grants from Nakatani Foundation. S.N. has received grants from Japan Research Foundation for Clinical Pharmacology, Naito Foundation, Takeda Science Foundation, Uehara Memorial Foundation, and Daiichi Sankyo Scholarship Donation Program within the past 3 years.

## Author contributions

Conception and design of study: K.K., S.T., D.S., S.N., G.R., A.G.G., and Y.H. Acquisition and analysis of data: K.K., S.T., and Y.H. Literature review and interpretation: K.K., S.T., D.S., S.N., Y.I., F.U., Y.T., J.T., and Y.H. Drafting the first manuscript or figures: K.K. Critical revision of the manuscript: K.K, S.T., D.S., S.N., Y.I., F.U., Y.T., J.T., F.C., W.M., E.T.C., Y.N., P.G., V.L., M.M., S.H., T.N., T.O., G.R., A.G.G., and Y.H. All of the authors contributed to and approved the final manuscript.

## Supporting information


**Figure S1.** Comparisons of whole‐brain local gyrification index (LGI) values between ultratreatment‐resistant schizophrenia (URS) and first‐line antipsychotics (FL‐Resp) and between treatment‐resistant schizophrenia (TRS) and non‐TRS. (a) Cluster showing significantly lower LGI in patients with URS compared with patients with schizophrenia who responded to FL‐Resp. (b) Cluster showing significantly lower LGI in patients with TRS compared with patients with schizophrenia who responded to non‐TRS (FL‐Resp). The maps are shown for the left hemispheres in the medial view. The horizontal bar shows cluster *P*‐value.

**Table S1.** Clusters with significant group differences in LGI
