# Supplementary figures and images for: Decreased cortical gyrification and surface area in the left medial parietal cortex in patients with treatment‐resistant and ultratreatment‐resistant schizophrenia
Source: Psychiatry Clin Neurosci. 2022 Oct 27;77(1):2–11. doi: 10.1111/pcn.13482 (PMC10092309; doi:10.1111/pcn.13482)

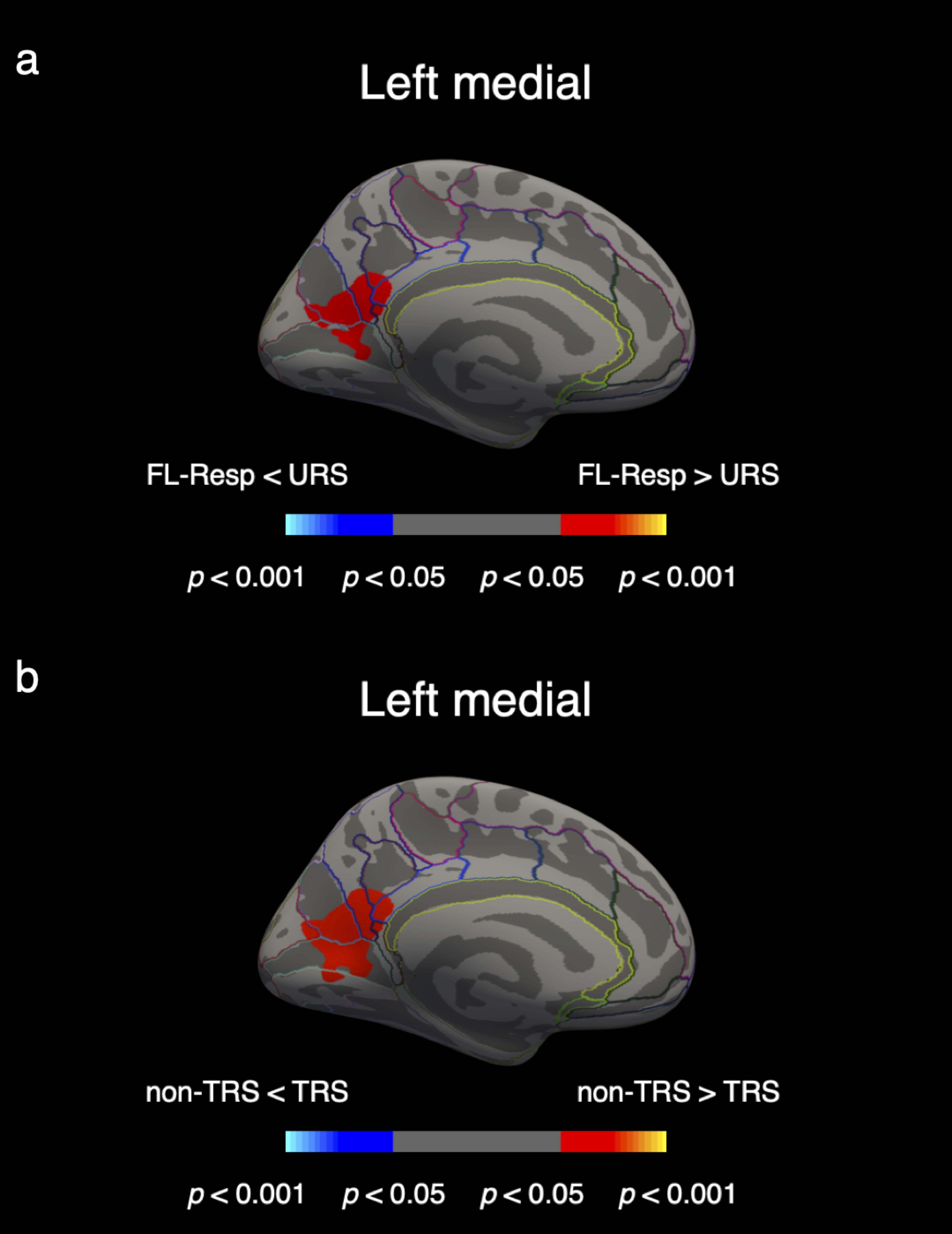

Supplement: Supplementary file 1 — Figure S1. Comparisons of whole‐brain local gyrification index (LGI) values between ultratreatment‐resistant schizophrenia (URS) and first‐line antipsychotics (FL‐Resp) and between treatment‐resistant schizophrenia (TRS) and non‐TRS. (a) Cluster showing significantly lower LGI in patients with URS compared with patients with schizophrenia who responded to FL‐Resp. (b) Cluster showing significantly lower LGI in patients with TRS compared with patients with schizophrenia who responded to non‐TRS (FL‐Resp). The maps are shown for the left hemispheres in the medial view. The horizontal bar shows cluster P‐value. [file PCN-77-2-s001.jpg]
