# Supplementary material for: Decreased cortical gyrification and surface area in the left medial parietal cortex in patients with treatment‐resistant and ultratreatment‐resistant schizophrenia
Source: Psychiatry Clin Neurosci. 2022 Oct 27;77(1):2–11. doi: 10.1111/pcn.13482 (PMC10092309; doi:10.1111/pcn.13482)
Supplement: Supplementary file 2 — Table S1. Clusters with significant group differences in LGI [file PCN-77-2-s002.pdf]

Table S1.

Clusters with significant group differences in local gyrification index.

	Cluster	Cluster size (mm^2)	MNI coordinates x	y	z	Cluster-wise *p*	Anatomical region
**a**	FL-Resp > URS	1231.21	-6.4	-58.4	20.8	0.01395	Left precuneus, parieto-occipital sulcus, cuneus, calcarine sulcus, subparietal sulcus, posterior-dorsal part of the cingulate gyrus, posterior-ventral part of the cingulate gyrus
**b**	non-TRS >TRS	1774.71	-6.4	-58.5	21.2	0.00599	Left precuneus, parieto-occipital sulcus, cuneus, calcarine sulcus, subparietal sulcus, posterior-dorsal part of the cingulate gyrus, posterior-ventral part of the cingulate gyrus, lingual gyrus

FL-Resp, schizophrenia patients who responded to first-line antipsychotics; MNI, Montreal Neurological Institute; TRS, patients with treatment-resistant schizophrenia; URS, patients with ultra-treatment-resistant schizophrenia.
